# Clinical Implications of Nonbiological Factors With Colorectal Cancer Patients Younger Than 45 Years

**DOI:** 10.3389/fonc.2021.677198

**Published:** 2021-07-07

**Authors:** Qi Liu, Ruoxin Zhang, Qingguo Li, Xinxiang Li

**Affiliations:** ^1^ Department of Colorectal Surgery, Fudan University Shanghai Cancer Center, Shanghai, China; ^2^ Department of Oncology, Shanghai Medical College, Fudan University, Shanghai, China

**Keywords:** non-biological factors, colorectal cancer, young, screening, prognosis

## Abstract

**Background:**

To evaluate the clinical implications of non-biological factors (NBFs) with colorectal cancer (CRC) patients younger than 45 years.

**Methods:**

In the present study, we have conducted Cox proportional hazard regression analyses to evaluate the prognosis of different prognostic factors, the hazard ratios (HRs) were shown with 95% confidence intervals (CIs). Kaplan–Meier method was utilized to compare the prognostic value of different factors with the log-rank test. NBF score was established according to the result of multivariate Cox analyses.

**Results:**

In total, 15129 patients before 45 years with known NBFs were identified from the SEER database. Only county-level median household income, marital status and insurance status were NBFs that significantly corelated with the cause specifical survival in CRC patients aged less than 45 years old (P < 0.05). Stage NBF 1 showed 50.5% increased risk of CRC-specific mortality (HR = 1.505, 95% CI = 1.411-1.606, P < 0.001). Stage NBF 0 patients were associated with significantly increased CRC-specific survival (CCSS) when compared with the stage NBF 1 patients in different AJCC TNM stages.

**Conclusions:**

NBF stage (defined by county-level median household income, marital status and insurance status) was strongly related to the prognosis of CRC patients. NBFs should arouse enough attention of us in clinical practice of patients younger than 45 years.

## Introduction

Colorectal cancer (CRC) is one of the most common malignant tumors. The vast majority of patients with CRCs are > 50 years of age. 75% of CRC patients present with rectal cancer and 80% with colon cancer at an age higher than 60 years at the time of diagnosis (1). However, the incidence rate of CRC is increasing in young persons, the American Cancer Society (ACS) therefore recommends average-risk CRC screening at 45 years old (2).

CRC incidence rates have risen by 1.3% and 2.3% per year in patients at the age of 40–49 years in the United States over the last two decades, respectively. On the contrast, incidence rates of patients over the age of 55 years have decreased by 2- to 3-fold, which is largely attributed to the screening of this disease (3).

Recently, the ACS recommended average-risk CRC screening in adults aged ≥ 45 years with stool-based test or a visual examination (4). It is worth noting that CRC screening before the age of 45 is still somewhat neglected, which may cause the increasing percentage of CRC patients aged less than 45 years.

The oncological outcomes of cancer patients would be affected by biological factors and non-biological factors (NBFs). The prognostic effects of different biological factors on CRC patients have been widely studied, including patient age, race, histological type, lymph node invasion, tumor grade, tumor size, gender and so on. The associations of NBFs with tumors, such as CRC, breast cancer and testicular cancer, have been reported (5–11). However, their prognostic significance was neglected to some extent (12–15).

Moreover, the widely utilized AJCC staging system is only based on the biological factors, and it is sometimes unable to accurately predict the prognosis of CRC patients. We therefore conducted this study to evaluate the implications of NBFs with staging, prognosis and clinical management of CRC patients younger than 45 years.

## Methods

### Patients

**Figure 1:**
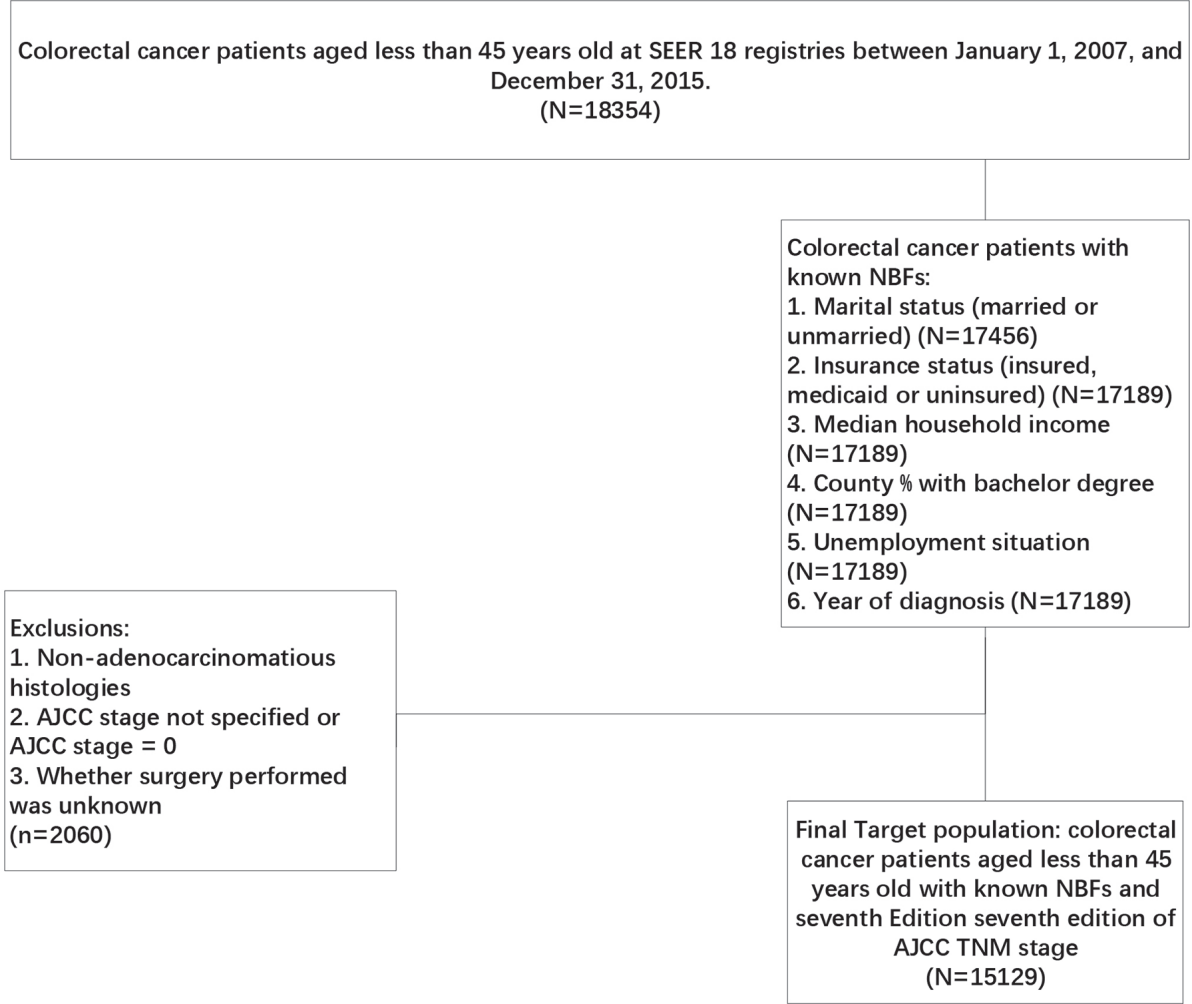
Flow diagram of patient selection.

The SEER-Stat software (SEER*Stat 8.3.8, https://seer.cancer.gov/seerstat/) was used in the present study, patients meeting the strict criteria were identified from the Surveillance, Epidemiology and End Results (SEER) database, which is a comprehensive source of population-based information on clinicopathological features and survival of cancer patients in the USA. Initially, CRC patients aged less than 45 years old were selected from SEER 18 registries between January 1, 2007 and December 31, 2015. Subsequently, only CRC patients with known NBFs were included in the present study according to the following criteria: ①. Marital status (married or unmarried), ②. insurance status (insured, medicaid or uninsured), ③. median household income, ④. county % with bachelor degree (N=17189), ⑤unemployment status (N=17189), ⑥. year of diagnosis (N=17189). In addition, patients with incomplete surgery history data, non-adenocarcinomatous histologies, non-specified AJCC stage and not specified or AJCC stage = 0 were excluded from our analyses (**Figure 1**). The primary endpoint of this study was CRC-specific survival (CCSS). The death of CRC patients was categorized as CRC-specific or non-CRC-related. CCSS of CRC-specific death was calculated from the date of diagnosis to the date of CRC death, whereas non-CRC related deaths were censored at the date of death.

### NBF Score, NBF Stage, and Statistical Analysis

Initially, univariate Cox analysis was conducted to identify all the independent prognostic variables. Subsequently, the prognostic factors with P value < 0.2 in the univariate analysis were entered into the multivariate Cox analyses, including gender, tumor grade, AJCC stage, surgery status, histology, the receipt of chemotherapy and all the NBFs (insurance status, county-level median household income, county % were unemployed, year of diagnosis, county % with bachelor degree and marital status), which indicated that only the variables county-level median household income, marital status and insurance status were significantly associated with the cause specific survival in patients before 45 years.

**Figure 2:**
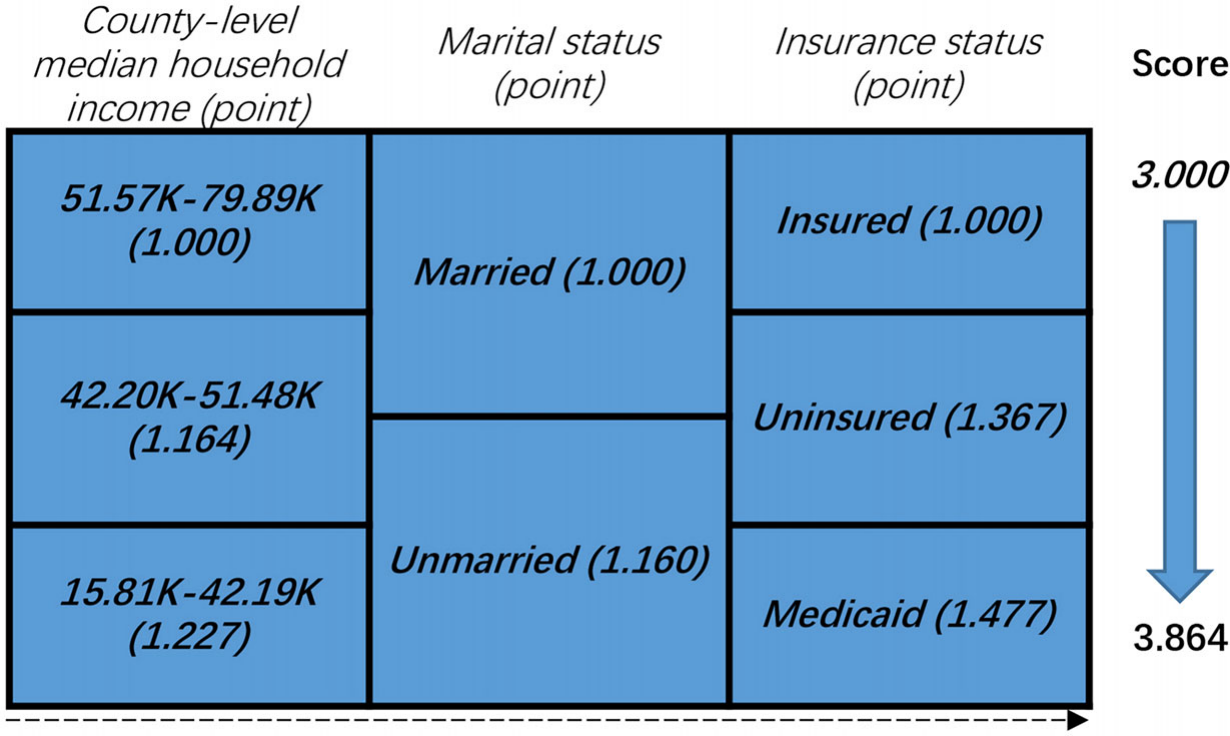
Non-biological factor (NBF) score in colorectal cancer patients younger than 45 years: risk-stratifications.

**Figure 3:**
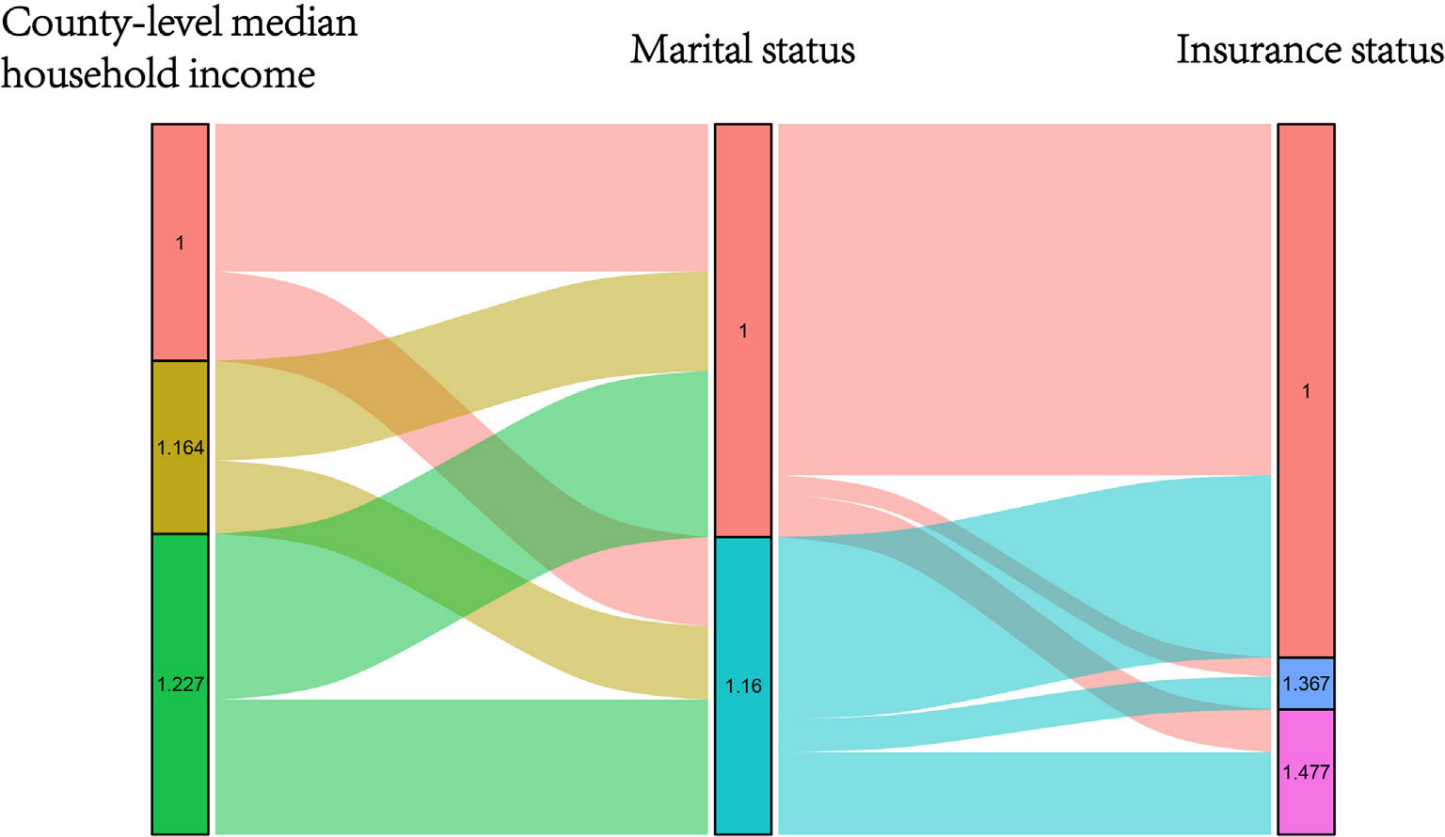
Graphical summary of the distribution and associations of different score subgroups in county-level median household income, insurance status and marital status, respectively.

The NBF score was determined according to the results of the multivariate Cox analysis. As shown in **Figure 2**, we considered the point of each group of each NBF equivalent to the value of the hazard ratios which were generated in multivariate Cox analysis. Subsequently, we assigned each patient a NBF score that was the total of the hazard ratio points in the three NBFs. For instance, a married and insured patient whose county-level median household income was 42.20–51.48 K (dollars) had a calculated score of the sum of “1.000”, “1.000”, and “1.164” which was equivalent to “3.164”. The NBF stage of each patient was subsequently stratified according to the NBF score. It was shown that the total score ranged from 3.000–3.864, which was divided into two groups with the median NBF score of all the CRC patients aged less than 45 years old as the cut-off value (3.227). Patients with lower NBF score were assigned to stage NBF 0 and others with higher NBF score were assigned to NBF 1 (14). The distribution and associations of county-level median household income, marital status and insurance status are presented in **Figure 3**.

In the present study, Cox proportional hazard regression models were constructed to evaluate the prognosis of different prognostic factors. The hazard ratios (HRs) were shown with 95% confidence intervals (CIs). Kaplan–Meier method was utilized to compare the prognostic value of different factors with the log-rank test. Only P-values lower than 0.05 were considered to reach statistical significance. Statistical analyses in the present were performed with the Statistical Package for Social Science (SPSS version 23; IBM Corp, Armonk, NY, USA).

## Results

In total, 15,129 patients were identified from the SEER database before 45 years with known NBFs. The median follow-up time was 41 (range, 0–119) months. A total of 3,730 (24.7%) patients succumbed to CRC at the end of the follow-up time. The baseline characteristics of the total cohort were summarized, as shown in **Table 1**.

**Table 1:** Baseline characteristics of colon cancer patients included in our study.

Characteristic	No. (%)
**Gender**	
Male	7947 (52.5)
Female	7182 (47.5)
**Tumor grade**	
Grade I	1001 (6.6)
Grade II	9915 (65.5)
Grade III	2692 (17.8)
Grade IV	425 (2.8)
Unknown	1096 (7.2)
**AJCC stage**	
I	2471 (16.3)
IIA	2854 (18.9)
IIB	400 (2.6)
IIC	208 (1.4)
IIIA	614 (4.1)
IIIB	2916 (19.3)
IIIC	2181 (14.4)
IVA	2313 (15.3)
IVB	1172 (7.7)
**Surgery**	
Surgery not performed	1125 (7.4)
Surgery performed	14004 (92.6)
**Histology**	
Adenocarcinoma	13483 (89.1)
Mucinous adenocarcinoma	1306 (8.6)
Signet-ring cell carcinoma	340 (2.2)
**Chemotherapy**	
No/unknown	4865 (32.2)
Yes	10264 (67.8)
**County % with bachelor degree**	
6.83%-26.58%	5068 (33.5)
26.62%-35.68%	5039 (33.3)
35.83%-54.45%	5022 (33.2)
**County-level median household income** ^#^	
15.81K-42.19K	6406 (42.3)
42.20K-51.48K	3685 (24.4)
51.57K-79.89K	5038 (33.3)
**County % were unemployed**	
1.29%-5.97%	5075 (33.5)
5.98%-7.80%	6201 (41.0)
7.84%-17.16%	3853 (25.5)
**Year of diagnosis**	
2007	1624 (10.7)
2008	1656 (10.9)
2009	1707 (11.3)
2010	1665 (11.0)
2011	1651 (10.9)
2012	1607 (10.6)
2013	1629 (10.8)
2014	1858 (12.3)
2015	1732 (11.4)
**Insurance status**	
Insured	11356 (75.1)
Medicaid	2664 (17.6)
Uninsured	1109 (7.3)
**Marital status**	
Married	8972 (58.1)
Unmarried	6337 (41.9)

**^#^**Shown in US dollars.

### NBFs Are Significant Prognostic Factors of Patients Before 45 years

As shown in **Table 2**, univariate Cox analyses resulted in the identification of the patient characteristics with P values less than 0.20. These data were introduced in multivariate Cox analyses. Only county-level median household income, marital status and insurance status were NBFs that were significantly associated with cause-specific survival in CRC patients aged less than 45 years old (P < 0.05). In addition, gender, tumor grade, AJCC stage, surgical status, histology and the receipt of chemotherapy were also found to be independent prognostic factors in CRC patients aged less than 45 years old. The variables including lower county-level median household income, Medicaid, uninsured and unmarried were found to be associated with higher risk of CRC-specific mortality (P < 0.01).

**Table 2:** Univariate and multivariable Cox regression analyses of all independent prognostic factors in patients before the recommended initiating colorectal cancer screening age.

Groups	Variable	Univariate analyses		Multivariate analyses	
		HR (95%CI)	*P*	HR (95%CI)	*P*
**Gender**			<0.001		<0.001
	**Male**	Reference		Reference	
	**Female**	0.864 (0.810-0.922)		0.845 (0.792-0.902)	
**Tumor grade**			<0.001		<0.001
	**Grade I**	Reference		Reference	
	**Grade II**	1.344 (1.141-1.582)		1.306 (1.106-1.542)	
	**Grade III**	3.133 (2.647-3.708)		2.255 (1.898-2.680)	
	**Grade IV**	3.222 (2.579-4.026)		2.158 (1.720-2.709)	
	**Unknown**	2.608 (2.160-3.148)		1.550 (1.279-1.879)	
**AJCC stage**			<0.001		<0.001
	**I**	Reference		Reference	
	**IIA**	1.761 (1.389-2.232)		1.674 (1.317-2.128)	
	**IIB**	5.186 (3.876-6.938)		4.485 (3.334-6.034)	
	**IIC**	5.966 (4.048-8.794)		5.252 (3.543-7.784)	
	**IIIA**	1.939 (1.380-2.724)		2.076 (1.468-2.935)	
	**IIIB**	4.279 (3.455-5.300)		4.116 (3.286-5.155)	
	**IIIC**	8.031 (6.516-9.897)		7.098 (5.687-8.859)	
	**IVA**	26.618 (21.784-32.525)		22.369 (18.059-27.707)	
	**IVB**	41.338 (33.597-50.862)		29.702 (23.749-37.148)	
**Surgery**			<0.001		<0.001
	**Surgery not performed**	Reference		Reference	
	**Surgery performed**	0.167 (0.154-0.182)		0.385 (0.351-0.423)	
**Histology**			<0.001		<0.001
	**Adenocarcinoma**	Reference		Reference	
	**Mucinous adenocarcinoma**	1.371 (1.233-1.524)		1.101 (0.987-1.227)	
	**Signet-ring cell carcinoma**	4.183 (3.632-4.818)		1.819 (1.567-2.112)	
**Chemotherapy**			<0.001		0.015
	**No/unknown**	Reference		Reference	
	**Yes**	2.677 (2.453-2.921)		0.888 (0.807-0.978)	
**County % with bachelor degree**			<0.001		0.072
	**6.83%-26.58%**	Reference		Reference	
	**26.62%-35.68%**	0.968 (0.897-1.045)		1.010 (0.924-1.103)	
	**35.83%-54.45%**	0.798 (0.737-0.864)		0.909 (0.815-1.015)	
**County-level median household income**			<0.001		0.001
	**51.57K-79.89K**	Reference		Reference	
	**42.20K-51.48K**	1.267 (1.161-1.382)		1.164 (1.052-1.288)	
	**15.81K-42.19K**	1.367 (1.267-1.476)		1.227 (1.103-1.365)	
**County % were unemployed**			<0.001		0.912
	**1.29%-5.97%**	Reference		Reference	
	**5.98%-7.80%**	1.160 (1.074-1.251)		0.996 (0.914-1.085)	
	**7.84%-17.16%**	1.267 (1.165-1.378)		0.979 (0.881-1.087)	
**Year of diagnosis**			0.458		
	**2007**	Reference			
	**2008**	1.049 (0.929-1.184)			
	**2009**	1,082 (0.960-1.221)			
	**2010**	0.985 (0.868-1.116)			
	**2011**	1.006 (0.885-1.143)			
	**2012**	1.021 (0.894-1.165)			
	**2013**	1.099 (0.958-1.260)			
	**2014**	0.957 (0.824-1.111)			
	**2015**	0.921 (0.762-1.113)			
**Insurance status**			<0.001		<0.001
	**Insured**	Reference		Reference	
	**Medicaid**	1.988 (1.842-2.146)		1.477 (1.363-1.600)	
	**Uninsured**	1.670 (1.493-1.869)		1.367 (1.219-1.534)	
**Marital status**			<0.001		<0.001
	**Married**	Reference		Reference	
	**Unmarried**	1.440 (1.350-1.536)		1.160 (1.084-1.241)	

### The NBF Stage Was Strongly Associated With the Prognosis of Patients Before 45 Years

A total of 8,830 (58.4%) patients were assigned to stage NBF 0 and 6,299 (41.6%) patients were assigned to stage NBF 1. Both univariate and multivariate Cox analyses indicated that NBF stage was a strong prognostic factor in CRC patients aged less than 45 years old, whereas stage NBF 1 was independently associated with 50.5% increased risk of CRC specific mortality (HR = 1.505, 95% CI = 1.411-1.606, P < 0.001; **Table 3**).

**Table 3:** Univariate and multivariable Cox regression analyses of NBF stage and other prognostic factors.

Groups	Variable	Univariate analyses		Multivariate analyses	
		HR (95%CI)	*P*	HR (95%CI)	*P*
**NBF-stage**			<0.001		<0.001
	**NBF-stage 0**	Reference		Reference	
	**NBF-stage 1**	1.725 (1.618-1.840)		1.505 (1.411-1.606)	
**Gender**			<0.001		<0.001
	**Male**	Reference		Reference	
	**Female**	0.864 (0.810-0.922)		0.851 (0.798-0.908)	
**Tumor grade**			<0.001		<0.001
	**Grade I**	Reference		Reference	
	**Grade II**	1.344 (1.141-1.582)		1.320 (1.118-1.559)	
	**Grade III**	3.133 (2.647-3.708)		2.271 (1.912-2.698)	
	**Grade IV**	3.222 (2.579-4.026)		2.171 (1.730-2.724)	
	**Unknown**	2.608 (2.160-3.148)		1.570 (1.296-1.901)	
**AJCC stage**			<0.001		<0.001
	**I**	Reference		Reference	
	**IIA**	1.761 (1.389-2.232)		1.689 (1.329-2.148)	
	**IIB**	5.186 (3.876-6.938)		4.591 (3.413-6.175)	
	**IIC**	5.966 (4.048-8.794)		5.331 (3.597-7.900)	
	**IIIA**	1.939 (1.380-2.724)		2.101 (1.486-2.972)	
	**IIIB**	4.279 (3.455-5.300)		4.167 (3.327-5.219)	
	**IIIC**	8.031 (6.516-9.897)		7.168 (5.743-8.946)	
	**IVA**	26.618 (21.784-32.525)		22.745 (18.364-28.170)	
	**IVB**	41.338 (33.597-50.862)		30.075 (24.050-37.611)	
**Surgery**			<0.001		<0.001
	**Surgery not performed**	Reference		Reference	
	**Surgery performed**	0.167 (0.154-0.182)		0.383 (0.349-0.420)	
**Histology**			<0.001		<0.001
	**Adenocarcinoma**	Reference		Reference	
	**Mucinous adenocarcinoma**	1.371 (1.233-1.524)		1.109 (0.994-1.236)	
	**Signet-ring cell carcinoma**	4.183 (3.632-4.818)		1.886 (1.625-2.189)	
**Chemotherapy**			<0.001		0.011
	**No/unknown**	Reference		Reference	
	**Yes**	2.677 (2.453-2.921)		0.884 (0.803-0.973)	

### Prognostic Significance of NBF Stage Following the Combination With TNM Stage

After the combination with NBF stage, each AJCC TNM stage was assigned to stage NBF 0 or stage NBF 1, including I NBF0, I NBF1, IIA NBF0, IIA NBF1, IIB NBF0, IIB NBF1, IIC NBF0, IIC NBF1, IIIA NBF0, IIIA NBF1, IIIB NBF0, IIIB NBF1, IIIC NBF0, IIIC NBF1, IVA NBF0, IVA NBF1, IVB NBF0 and IVB NBF1.

**Figure 4:**
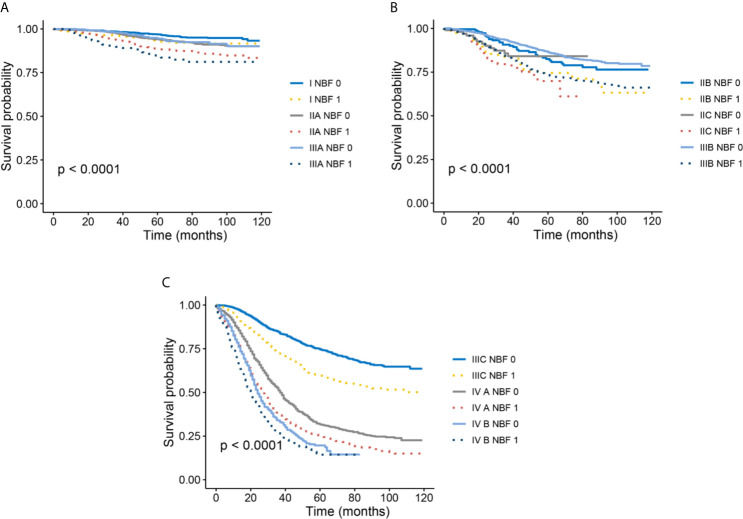
Kaplan–Meier survival curves of NBF-TNM staging system. **(A)** Cancer-specific survival (CSS) of I-NBF 0 stage, I- NBF 1 stage, IIA-NBF 0 stage, IIA- NBF 1 stage, IIIA- NBF 0 stage, and IIIA- NBF 1 stage. **(B)** CSS of IIB- NBF 0 stage, IIB- NBF 1 stage, IIC- NBF 0 stage, IIC- NBF 1 stage, IIIB- NBF 0 stage, and IIIB- NBF 1 stage. **(C)** CSS of IIIC- NBF 0 stage, IIIC- NBF 1 stage, IVA- NBF 0 stage, IVA- NBF 1 stage, IVB- NBF 0 stage, and IVB- NBF 1 stage.

Kaplan-Meier survival analyses indicated that all stage NBF 0 patients were associated with a statistically significant increased CCSS compared to stage NBF 1 patients in different AJCC TNM stages (**Figures 4A–C**). Moreover, these results were also validated in multivariate Cox analyses as follows: All the stage NBF 0 patients indicated lower HRs compared with the respective stage NBF 1 patients, which was in agreement with the results of the Kaplan-Meier survival analyses.

It should also be noted that several stage NBF 1-TNM patients exceeded stage NBF 0 with higher conventional AJCC TNM stage. For example, the risk of CRC-specific mortality of stage IIB NBF 1 (HR = 4.264, 95%CI = 2.843-6.396, using stage NBF 1 as the reference, P < 0.001) was significantly higher than that of stage IIC NBF 0 (HR = 2.988, 95%CI = 1.611-5.541, using stage NBF 1 as the reference, P = 0.001). The risk of CRC-specific mortality of stage IIC NBF 1 (HR = 5.095, 95%CI = 3.034-8.556, using stage NBF 1 as the reference, P < 0.001) was significantly higher than that of stage IIIA NBF 0 (HR = 0.967, 95%CI = 0.580-1.612, using stage NBF 1 as the reference, P = 0.898) and the risk of CRC-specific mortality of stage IIIA NBF 1 (HR = 2.556, 95%CI = 1.586-4.121, using stage NBF 1 as the reference, P < 0.001) was significantly higher than that of stage IIIB NBF 0 (HR = 2.191, 95%CI = 1.584-3.030, using stage NBF 1 as the reference, P < 0.001). Finally, the risk of CRC-specific mortality of stage IIIB NBF 1 (HR = 4.327, 95%CI = 3.146-5.952, using stage NBF 1 as the reference, P < 0.001) was significantly higher than that of stage IIIC NBF 0 (HR = 4.119, 95%CI = 3.001-5.651, using stage NBF 1 as the reference, P < 0.001), which indicated that stage NBF 1 could increase the diagnostic value of conventional TNM stage (**Table 4**). In other words, the NBF stage could significantly affect the prognosis of patients younger than 45 years.

**Table 4:** Prognosis of NBF-stage and TNM stage in patients before the recommended initiating colorectal cancer screening age.

AJCC TNM staging system					TNM-C staging system				
Stage	Number of the patients	Cancer-specific survival			Stage	Number of the patients	Cancer-specific survival		
		HR (95% CI)	SE	*P* value			HR (95% CI)	SE	*P* value
**I**	2471	Reference	\	\	**I NBF0**	1647	0.562 (0.381-0.829)	0.198	0.004
					**I NBF1**	824	Reference	\	\
**IIA**	2854	1.689 (1.329-2.148)	0.122	<0.001	**IIA NBF0**	1702	0.941 (0.663-1.335)	0.178	0.733
					**IIA NBF1**	1152	1.668 (1.181-2.357)	0.176	0.004
**IIB**	400	4.591 (3.413-6.175)	0.151	<0.001	**IIB NBF0**	202	2.691 (1.718-4.215)	0.229	<0.001
					**IIB NBF1**	198	4.264 (2.843-6.396)	0.207	<0.001
**IIC**	208	5.331 (3.597-7.900)	0.201	<0.001	**IIC NBF0**	101	2.988 (1.611-5.541)	0.315	0.001
					**IIC NBF1**	107	5.095 (3.034-8.556)	0.264	<0.001
**IIIA**	614	2.101 (1.486-2.972)	0.177	<0.001	**IIIA NBF0**	415	0.967 (0.580-1.612)	0.261	0.898
					**IIIA NBF1**	199	2.556 (1.586-4.121)	0.244	<0.001
**IIIB**	2916	4.167 (3.327-5.219)	0.115	<0.001	**IIIB NBF0**	1713	2.191 (1.584-3.030)	0.165	<0.001
					**IIIB NBF1**	1203	4.327 (3.146-5.952)	0.163	<0.001
**IIIC**	2181	7.168 (5.743-8.946)	0.113	<0.001	**IIIC NBF0**	1223	4.119 (3.001-5.651)	0.161	<0.001
					**IIIC NBF1**	958	6.829 (4.985-9.356)	0.161	<0.001
**IVA**	2313	22.745 (18.364-28.170)	0.109	<0.001	**IVA NBF0**	1241	14.300 (10.554-19.374)	0.155	<0.001
					**IVA NBF1**	1072	19.819 (14.615-26.878)	0.155	<0.001
**IVB**	1172	30.075 (24.050-37.611)	0.114	<0.001	**IVB NBF0**	586	20.162 (14.712-27.630)	0.161	<0.001
					**IVB NBF1**	586	24.939 (18.215-34.146)	0.160	<0.001

## Discussion

The present study demonstrated that NBF stage was strongly related to the prognosis of patients before 45 years, whereas stage NBF 1 was independently associated with 50.5% increased risk of CRC specific mortality. Following combination with the TNM stage, the results demonstrated that NBF 0 patients were associated with a statistically significant increased CCSS compared to the stage NBF 1 patients in all the respective AJCC TNM stages. It should also be noted that several stages of NBF 1-TNM patients exceeded stage NBF 0 with higher conventional AJCC TNM stage. For example, the risk of CRC-specific mortality of stage IIB NBF 1 patients was significantly higher than that of stage IIC NBF 0 subjects, whereas the risk of CRC-specific mortality of stage IIC NBF 1 patients was significantly higher than that of stage IIIA NBF 0. The risk of CRC-specific mortality of stage IIIA NBF 1 subjects was significantly higher than that of stage IIIB NBF 0 subjects, whereas the risk of CRC-specific mortality of stage IIIB NBF 1 patients was significantly higher than that of stage IIIC NBF 0 patients, indicating that stage NBF 1 could increase the diagnostic value of the conventional TNM stage. In other words, the NBF stage could significantly affect the prognosis of patients younger than 45 years. Therefore, the present findings indicated that the combination of the NBF stage could increase the prognostic value of the TNM stage system. 45 years.

Incidence rates of patients over the age of 55 years have shown a decline during the last several decades. This trend was accelerated in 2000, and this phenomenon would be even more pronounced in adults aged 65 years or older (16). In contrast to these subjects, CRC incidence rates have increased by 1.3% and 2.3% per year in patients at the age of 40–49 years in the United States over the last two decades (3). The vast majority of CRCs occurred following the age of 50. Therefore, the ACS recommended average-risk CRC screening in adults aged ≥ 45 years with stool-based test or a visual examination. These findings indicate that CRC patients under the age of 45 are still somewhat ignored (4).

NBFs have been demonstrated to contribute to tumor development by previous studies. NBFs may act directly or indirectly to facilitate the consequences of different biological changes, thus affecting the prognostic effect of the biological factors in cancer patients (12).

The present study indicated that three NBFs were significantly associated with the oncological outcomes of CRC prior to 45 years, including county-level median household income, marital status and insurance status. The lower the income, the worse the prognosis of CRC (the income of 51.57K-79.89K was used as reference and the income of 15.81k-42.19K increased the risk of death by 22.7% compared with the income of 51.57K-79.89K). The prognostic effect of income on survival in the present study was in agreement with a previous study in ovarian cancer (17). This may be attributed to the fact that low-income patients were less likely to prefer active treatment owing to the fragile financial support network in CRC treatment.

As shown in our previous analyses, in the United States, Medicaid increased the risk of CRC-specific mortality death by 47.7% compared with that noted in insured patients. We held the view that late initiating treatment, inadequate treatment and poor physical conditions might contribute to the poor prognosis of young CRC patients with Medicaid. Previous studies have reported the prognostic effect of insurance status in many cancers, and Medicaid or uninsured patients would have worse survival compared with insured ones (18–21).

It has been reported in several previous studies that marital status had a prognostic effect on survival of several cancer types including rectal cancers (11, 15, 22–25). The improved prognosis noted in married CRC patients can be attributed to the improved endocrine, cardiovascular and immune function as well as treatment compliance in married patients (26).

Previous studies have proposed the inadequate prognostication of the present AJCC TNM staging system in CRC (27–29). Therefore, in the present study, the implications of NBFs with staging, prognosis and clinical management were assessed in CRC patients before 45 years.

The current study demonstrated that county-level median household income, marital status and insurance status were significantly associated with cause-specific survival in CRC patients younger than 45 years. NBFs were often neglected in clinical practice and patients with poor NBFs deserved more attention and a more intense treatment. The present study indicated that NBF stage was strongly related to the prognosis of patients. Following combination with the TNM stages, NBF 0 patients were associated with a statistically significant increased CCSS compared to the stage NBF 1 patients in all the respective AJCC TNM stages. Therefore, the present study findings indicated that the combination of the NBF stage could increase the prognostic value of the TNM stage.

The present study aimed to increase the information regarding CRC patients younger than 45 years, as well as analyze the NBFs that significantly affect the prognosis of CRC patients, including only county-level median household income, marital status and insurance status. NBFs should arouse sufficient attention of us in clinical practice of patients younger than 45 years. In such way, the research focus on these aspects will be enhanced by the scientific community.

Several limitations of this study should be addressed. Firstly, our analyses were only based on a US population. In the future, therefore, a validation study should be carried out. In the validation study, patients could be from countries beyond the United States. In addition, the validation study could investigate the prognostic value of NBFs in older CRC patients who were not included in the present study, which might lead to other interesting conclusions. And analyses could also be conducted based on different stratification factors such as gender, race and tumor stage. Above all, recruited patients should have complete information of NBFs including individual income, education, insurance, marital status and employment status. Secondly, some prognostic factors were not available in the SEER database and they were not included in our analyses, such as serum biomarkers, family history, microsatellite instability status, ras mutation and braf v600e status (30–32). Finally, the analyses were merely based on retrospective data, which would cause inevitable bias.

## Conclusion

County-level median household income, marital status and insurance status were significantly associated with cause-specific survival in CRC patients younger than 45 years. The present study showed that NBF stage was strongly related to the prognosis of patients. Following combination with the TNM stages, NBF 0 patients were associated with a statistically significant increased CCSS compared to the stage NBF 1 patients in all the respective AJCC TNM stages, indicating the combination of the NBF stage could increase the prognostic value of the TNM stage. Staging45 years NBFs should not be neglected in clinical practice and patients with poor NBFs deserved more attention and more intense treatment of CRC patients before 45 years.

## Data Availability Statement

Publicly available datasets were analyzed in this study. This data can be found here: https://seer.cancer.gov/data/access.html.

## Ethics Statement

The study was approved by the Ethical Committee and Institutional Review Board of the Fudan University Shanghai Cancer Center. The data did not include the use of human subjects or personal identifying information and no informed consent was required for this study.

## Author Contributions

QGL and XL conceptualized and designed the study. QL and RZ conducted the analyses of the study. QL interpreted the data. QL and RZ drafted the manuscript. QL revised the manuscript. All authors contributed to the article and approved the submitted version.

## Funding

This research was supported by the National Science Foundation of China (No. 81772599, 81972260 and 81702353) and Shanghai Municipal Natural Science Foundation (17ZR1406400). The funders had no role in the study design, data collection and analysis, decision to publish, or preparation of the manuscript.

## Conflict of Interest

The authors declare that the research was conducted in the absence of any commercial or financial relationships that could be construed as a potential conflict of interest.
